# Another Stultification, a Homœopathic Strike

**Published:** 1886-06

**Authors:** 


### ANOTHER STULTIFICATION—A HOMŒOPATHIC STRIKE.

The Southern Homœopathic Association, which is to Homœopathic State Associations what the American Medical Association is to the several State Medical Associations—their national onganization, met recently in New Orleans. Their "organ" (wind instrument) is jubilant over the fact—said to be a fact—that the "roll of members now numbers *fifty-seven.*!" (stand from under!!) and a delighted delegate writing from up north somewhere, to the "President"—the Austin Jeremy-Didler—who is to the Homœopathic lay-out what the fellow in Mikado was to the government—the Pooh-Bah—or everything "high"—declares on his honor that he actually "counted twenty-seven delegates on the floor at one time."—Great Scott! but wasn't that a gathering of the clans to make the average alopathic head swim! In a report of the proceedings of this mighty throng of mutual admirers published in said "organ" we find the solemn declaration in the shape of a resolution passed.

"That *this Association is opposed to State Medicine*".

Doubtless they were under the impression that by "State Medicine" is meant some kind of control of practice by the State, in the interest of the hated "Regulars;" hence, in a spirit of charity and commiseration for their ignorance and their fears we inform them—that by "State Medicine" is meant "public hygiene;" but for fear they do not know what that is, we will give it in plain English—in Prof. Chaille's own words.

"State Medicine is the application of the aggregate of medical knowledge to the public weal"—the *prevention of epidemics* —the protection of the public health from all kinds, and all sources of danger, (quacks included.)

Let it go on record that *twenty-seven of this class of persons* in these free United States have solemnly declared themselves, in convention assembled, opposed to the *protection of the public health*; opposed to *public sanitation*. "A little learning is" indeed "a dangerous thing." Gov. Ireland will have to call out the militia to quell this mob, when Dr. Cummings goes to lay those sewers in Austin, so much needed for the "protection of the public health."

